# The role of cisplatin in modulating the tumor immune microenvironment and its combination therapy strategies: a new approach to enhance anti-tumor efficacy

**DOI:** 10.1080/07853890.2024.2447403

**Published:** 2025-01-06

**Authors:** Guandu Li, Xiangyu Che, Shijin Wang, Dequan Liu, Deqian Xie, Bowen Jiang, Zunwen Zheng, Xu Zheng, Guangzhen Wu

**Affiliations:** a Department of Urology, the First Affiliated Hospital of Dalian Medical University, Dalian, Liaoning, China; b Department of Cell Biology, College of Basic Medical Science, Dalian Medical University, Dalian, Liaoning, China

**Keywords:** Cisplatin, tumor immune microenvironment, combination therapy, immune therapy, lipid metabolism disruptors

## Abstract

Cisplatin is a platinum-based drug that is frequently used to treat multiple tumors. The anti-tumor effect of cisplatin is closely related to the tumor immune microenvironment (TIME), which includes several immune cell types, such as the tumor-associated macrophages (TAMs), cytotoxic T-lymphocytes (CTLs), dendritic cells (DCs), myeloid-derived suppressor cells (MDSCs), regulatory T cells (Tregs), and natural killer (NK) cells. The interaction between these immune cells can promote tumor survival and chemoresistance, and decrease the efficacy of cisplatin monotherapy. Therefore, various combination treatment strategies have been devised to enhance patient responsiveness to cisplatin therapy. Cisplatin can augment anti-tumor immune responses in combination with immune checkpoint blockers (such as PD-1/PD-L1 or CTLA4 inhibitors), lipid metabolism disruptors (like FASN inhibitors and SCD inhibitors) and nanoparticles (NPs), resulting in better outcomes. Exploring the interaction between cisplatin and the TIME will help identify potential therapeutic targets for improving the treatment outcomes in cancer patients.

## 1. Introduction

Chemotherapy is the most widespread anti-tumor treatment strategy at present [1–4]. Till the 1960s, pure organic compounds were primarily used for anti-cancer chemotherapy [5]. Cisplatin, a platinum-based anti-tumor compound, was discovered in the 1960s [6] and greatly advanced tumor chemotherapy by offering more effective and diverse treatment options [7]. It has been particularly effective against bladder, ovarian, and breast cancers, among others [8,9]. Furthermore, almost 50% of cancer patients choose cisplatin for commencing chemotherapy [10], and the standard first-line treatment for most cancers, especially metastatic uroepithelial cancer [11] and bladder cancer [12], is based on the combination of cisplatin with other agents. In fact, cisplatin remains the mainstay of systemic cancer therapies despite advances in targeted and immunogenic treatments [13]. However, the therapeutic efficacy of cisplatin is limited due to its toxicity against liver, heart, cochlear, and peripheral nerve cells [14].

The tumor immune microenvironment (TIME) consists of various cell types that either promote or hinder the growth of tumors, including innate immune cells like macrophages, neutrophils, natural killer (NK) cells, dendritic cells (DCs) and myeloid-derived suppressor cells (MDSCs), and the adaptive T cells and B cells. In addition, extracellular immune factors and cell-surface molecules are also components of TIME [15–19]. Based on the relative proportion of the different immune cells, TIME can be categorized into the infiltration-exclusion (I-E), infiltration-inflammation (I-I), and tertiary lymphoid structure (TLS) types [19]. Interestingly, the type of TIME can influence the response to cisplatin [20,21]. While tumor cells have evolved strategies to evade immune surveillance and attack by altering the TIME [22], the immune cells within the tumors can also induce resistance to cisplatin through various mechanisms [23].

Several preclinical studies have shown that combining cisplatin with other therapeutic agents can overcome the resistance of tumor cells to the drug. For example, the combination of cisplatin with immune checkpoint blockers and lipid metabolism inhibitors can sensitize tumor cells to cisplatin by altering the function of intra-tumoral immune cells [24,25]. Inhibitors targeting immune checkpoint molecules such as programmed death receptor 1 (PD-1), programed death ligand 1 (PD-L1) and cytotoxic T lymphocyte-associated protein 4 (CTLA-4) block the co-inhibitory signals in immune-reactive T cells [26,27], thereby reversing the immunosuppressive TIME and enhancing immune-mediated clearance of tumor cells [28].

In this review, we have described the impact of cisplatin on the TIME, and discussed the underlying mechanisms of cisplatin resistance involving the TIME. Furthermore, the current challenges of cisplatin therapy and possibilities of combination therapies have also been discussed.

## 2. Mechanism of action of cisplatin

**Figure 1.**
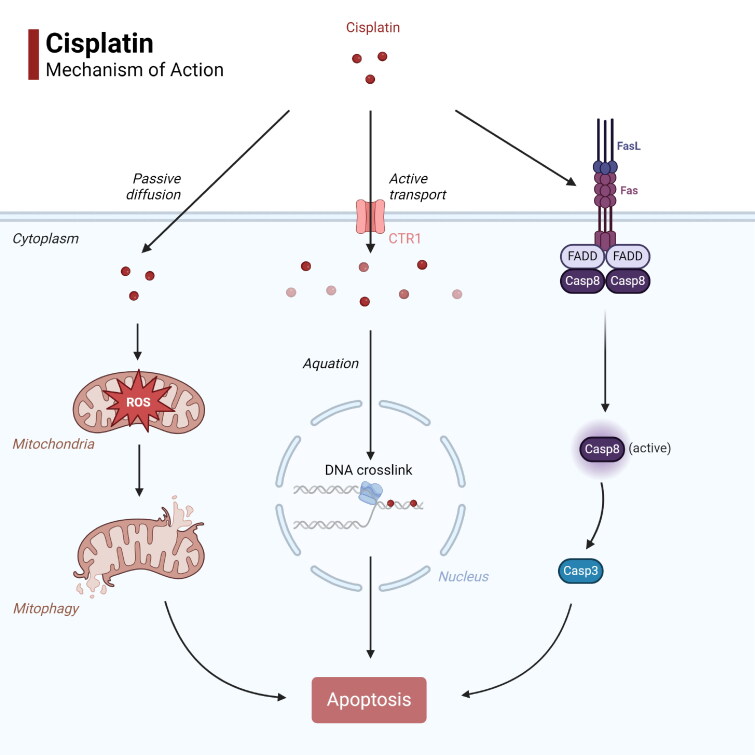
Mechanism of action for cisplatin. (1) Cisplatin enters tumor cells *via* CTR1 or passive diffusion. Cisplatin enters the nucleus and cross-links with DNA molecules, leading to apoptosis. (2) Cisplatin leads to mitophagy through the production of ROS, which leads to apoptosis. (3) Cisplatin activates the Fas receptor pathway, which activates Casp8 downstream, thereby stimulating Casp3 and promoting apoptosis. CTR1: copper transporter 1, ROS: reactive oxygen species, Casp3: caspase 3, Casp8: caspase 8, FasL: Fas ligand, FADD: Fas-associated death domain. Created with BioRender.com.

The cytotoxic effect of cisplatin is associated with its ability to form DNA adducts that inhibit DNA replication and repair [29,30]. Cisplatin enters tumor cells *via* copper transporter 1, and is activated by the cytoplasmic water molecules or other small sulfhydryl-containing molecules [31]. Following nuclear translocation, cisplatin creates intra- and interstrand crosslinks in the DNA molecules. The resulting damage in DNA structure [32] triggers cell cycle arrest and apoptosis in the rapidly proliferating tumor cells [33] (Figure 1).

Intracellular oxidative stress is a potential mechanism through which chemotherapeutic agents promote cell death [34]. Cisplatin can induce oxidative stress *via* reactive oxygen species (ROS), such as hydroxyl radicals and superoxide, in a concentration- and time-dependent manner [35]. Since mitochondria is the primary site of ROS production, cisplatin-induced oxidative stress often leads to abnormal mitochondrial calcium uptake, mitochondrial membrane depolarization and decreased ATP synthesis, eventually activating the apoptotic pathway [1,36](Figure 1). In addition, preclinical studies have shown that reducing intracellular glutathione (GSH) biosynthesis can sensitize gastric cancer cells to cisplatin by increasing oxidative stress [37].

Fas ligand (FasL) is a cell surface protein that regulates programmed cell death [38]. Fas-FasL binding activates the caspase pathway and initiates apoptosis [38]. Cisplatin can induce apoptosis in cancer cells by disrupting the plasma membrane and activate the Fas death receptor pathway [39] (Figure 1). Preclinical studies have shown that Fas overexpression can sensitize small cell lung cancer cells to cisplatin-induced apoptosis [40]. In addition, cytoplasmic acidification [4]^1^ and estrogen receptor (ER) stress [4]^2^ have also been identified as potential modes of cisplatin action.

## 3. Effect of cisplatin on TIME

**Figure 2.**
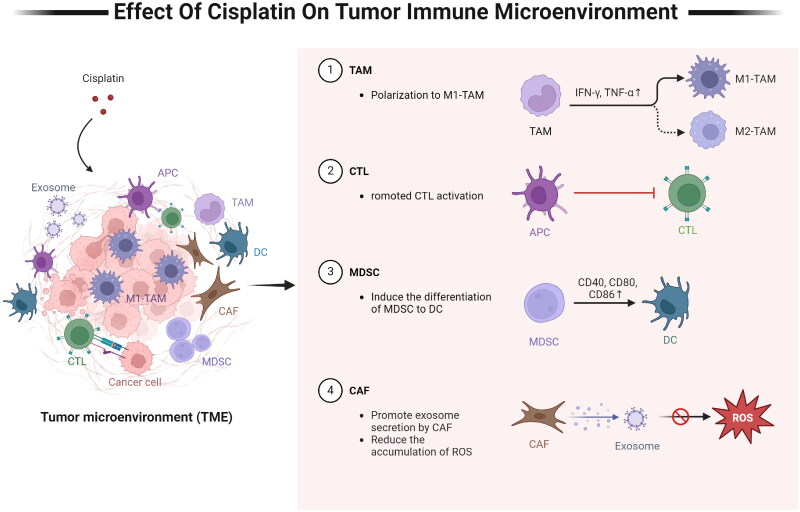
Effect of cisplatin on the TIME. (1) Cisplatin promotes M1 polarization of TAMs by increasing the secretion of IFN-γ and TNF-α. (2) Cisplatin treatment of tumor cells produces type I IFN, which upregulates costimulatory molecules on APCs and promotes the activation of CTLs.(3) cisplatin potentially induces the differentiation of MDSCs into DCs by increasing the expression of DC markers such as CD40, CD80, and CD86.(4) cisplatin promotes exosome secretion by CAFs, which inhibits ferroptosis in tumor cells by reducing the accumulation of ROS, thereby increasing resistance to cisplatin. IFN-γ: interferon-γ, TNF-α: tumor necrosis factor-α, TAM: tumor-associated macrophage, APC: antigen-presenting cell, CTL: cytotoxic T-lymphocyte, MDSC: myeloid-derived suppressor cell, DC: dendritic cell, CAF: cancer-associated fibroblasts, ROS: reactive oxygen species. Created with BioRender.com.

The TIME is a key determinant of tumor development and the response to chemotherapy [43]. Alterations in TIME can significantly enhance the anti-tumor effects of cisplatin and improve patient prognosis [44]. Recent evidence have shown that cisplatin can inhibit cancer progression by activating anti-tumor immune responses [45]. This contradicts the well-documented myelosuppressive effects of most chemotherapeutic drugs, including cisplatin [45]. Nonetheless, cisplatin is known to regulate the function of immune cells related to tumor progression, including tumor-associated macrophages (TAMs), CTLs, and DCs (Figure 2). A greater understanding the effects of cisplatin on these immune cells can provide a basis for designing strategies to overcome drug resistance and enhance therapeutic efficacy [46]. The effects of cisplatin on the different immune cells in discussed in the following section.

### 3.1. Tumor-associated macrophages (TAMs)

TAMs are the primary immune cells in the tumor microenvironment, and secrete inflammatory factors and cytokines that influence tumor progression [47,48]. The TAMs are classified into the morphologically and functionally distinct M1-like and M2-like phenotypes [49]. M1-like macrophages are smaller and rounder, while M2-like macrophages are larger and longer [50]. McWhorter et al. successfully polarized murine bone marrow-derived macrophages to the elongated M2 phenotype by culturing the cells on a micropatterned groove measuring 20 µm in width [51]. Functionally, M1-like TAMs promote anti-tumor immune responses, whereas M2-like TAMs promote tumor growth by inducing an immunosuppressive microenvironment [52]. M1-like macrophages are activated by tumor necrosis factor-α (TNF-α), interleukin-1 β (IL-1β) and interferon-γ (IFN-γ), and express pro-inflammatory markers such as IL-1, IL-6, CD68, CD80, inducible nitric oxide synthase (iNOS) and major histocompatibility complex-II (MHC-II) [53]. In contrast, the M2-like macrophages are typically characterized by low expression of MHC-II and high expression of scavenger receptor CD163, and release immunosuppressive cytokines like vascular endothelial growth factor (VEGF) and arginase 1 (Arg-1), IL-10, transforming growth factor-β (TGF-β) and indoleamine 2,3-dioxygenase (IDO) [54].

Cisplatin can reverse the immunosuppressive conditions in the tumor microenvironment by promoting M1 polarization of the TAMs [55]. Furthermore, cisplatin triggers immunogenic cell death (ICD), which in turn enhances the antigenicity of tumor cells and the efficacy of M1-like TAMs. Preclinical studies have shown that cisplatin promotes the infiltration and activation of the TAMs by upregulating CD62L and CD301 [22]. Furthermore, cisplatin also increases production of cytokines like IFN-γ, TNF-α and IL-6, which in turn recruit M1-like TAMs to the tumor site [56]. Interestingly, M1-like TAMs secrete exosomes that can encapsulate cisplatin and transport the drug to the tumor cells, resulting in enhanced anti-tumor effects. Likewise, cisplatin also binds to the exosomes secreted by M1-like TAMs, and upon internalization into the tumor cells, activates the Bax/Caspase-3 apoptosis cascade [57]. However, the exosomes secreted by M2 macrophages upregulate miR-3679-5p in the tumor cells and inhibit the secretion of the regulatory factor NEDD4L, which decreases the stability of c-Myc and desensitizes the tumor cells to cisplatin [58]. Apart from these two methods, an additional preclinical study showed that cisplatin upregulates the expression of SYT11 (a non-Ca2+ binding synaptic binding protein) and inhibits TAM endocytosis in breast cancer patients, which reduces TAM clearance by tumor cells, a key factor limiting tumor cell sensitivity to cisplatin [59].

### 3.2. Cytotoxic T lymphocytes (CTLs)

CTLs are the key immune effectors that recognize and clear tumor cells [60]. The primary T cells differentiate into CTLs and memory T cells in the peripheral lymphoid organs [61]. CTLs recognize and rapidly eliminate tumor cells bearing specific antigens, while the memory cells provide long-term immunity [62]. Cisplatin can enhance CTLs infiltration and activation by initiating co-stimulatory signals, thereby enhancing anti-tumor immune responses [63]. Furthermore, cisplatin-induced DNA damage in tumor cells [6]^4^ generates double-stranded DNA (dsDNA) fragments [65], which trigger the release of CCL5 and CXCL10 *via* activation of the cGAS-STING signaling pathway [66]. These chemokines promote the infiltration and activation of CTLs, eventually enhancing therapeutic efficacy [67]. Studies show that the cGAS-STING pathway plays key roles in immune responses as well as tumor development [68–72]. In one preclinical study, cisplatin triggered apoptosis of Lewis lung carcinoma cells in the presence of anti-Fas antibody by upregulating the surface expression of Fas, which further enhanced CTLs infiltration and activation [73]. Cisplatin can also activate CTLs by promoting infiltration of antigen-presenting cells (APCs) [74], and increasing production of type I IFN and IL-6 [65]. Furthermore, cisplatin contributes to the recruitment and activation of CTLs by upregulating Ariadne RBR E3 ubiquitin protein ligase 1 (ARIH1) [63], chemokine ligand 20 (CCL20) and IL-1β in the tumor tissues [75]. Therefore, elucidating the specific mechanism through which cisplatin activates anti-tumor CTLs could provide a basis for improving therapeutic effects.

### 3.3. Myeloid-derived suppressor cells (MDSCs)

MDSCs are a specialized subset of myeloid regulatory cells (MRCs) that play a key role in establishing an immunosuppressive milieu in the tumors [76]. MDSCs can be classified as monocytic MDSCs (M-MDSCs) and polymorphonuclear MDSCs (PMN-MDSCs) based on surface markers and morphology [77]. Human PMN-MDSCs are characterized as CD11b + CD14-CD15+/CD66b+, and M-MDSCs as CD11b + CD14 + HLA-DR^low^ [78]. MDSCs inhibit the anti-tumor activity of T cells through Arg-1, iNOS, ROS, and the anti-inflammatory cytokine IL-10 [79].

Preclinical studies have shown that the combination of α-Gr1 and α-Ly6C antibodies targeting PMN-MDSCs and M-MDSCs in cisplatin-resistant bladder cancer cells significantly reduced tumor volume and increased the infiltration of CD8+ T cells [77]. Another preclinical study showed that cisplatin selectively reduced the abundance of MDSCs in melanoma tissues, and attenuated their immunosuppressive effects [80]. Furthermore, cisplatin reduced the expression of Gr-1 on MDSCs and increased the expression of DC markers like CD40, CD80 and CD86, indicating that cisplatin can potentially induce the differentiation of immunosuppressive MDSCs to the immunostimulatory DCs [80]. A study conducted on melanoma and head and neck squamous cell carcinoma patients found that cisplatin significantly reduced the ability of M-MDSCs to inhibit T cell responses *in vitro* by downregulating COX-2 and arginase-1 *via* STAT3 blockade [81]. Cisplatin also reduced the number of MDSCs in bladder tumors by inhibiting methyltransferase 3 (METTL3)-mediated methylation of granulocyte colony-stimulating factor (G-CSF) [82]. Taken together, cisplatin can augment the effects of anti-tumor immunotherapy by inhibiting M-MDSCs.

### 3.4. Other immune cells

Cisplatin can promote the infiltration and activation of various immune cells in the TIME and enhance patient outcomes. DCs are the primary APCs that initiate anti-tumor immune responses by phagocytosing and cross-presenting tumor antigens to T lymphocytes [83]. Preclinical studies have shown that cisplatin can stimulate adaptive immune responses by inducing ICD, which releases a number of stress/risk signals that recruit and activate DCs [55]. Cisplatin enhanced the efficacy of chemotherapy and immunotherapy against cervical cancer cells through inactivation of the JAK1-STAT3 signaling pathway [84]. Inhibition of the JAK1-STAT3 signaling pathway blocked the expression of key glycolytic enzymes, which further facilitated ICD induction in hepatocellular carcinoma, thereby promoting DC activation, enhancing anti-tumor immune responses and improving patient prognosis [85]. In addition, cisplatin can enhance the infiltration and activation of DCs by upregulating IL-10, thus further activating a robust anti-tumor immune response and improving treatment outcomes [86]. In addition, cisplatin activates NK cells, a class of intrinsic lymphoid cells that spontaneously kill target cells and are the primary innate immune effectors against tumors [87]. Preclinical studies have shown that cisplatin inhibits the androgen receptor (AR) in hepatocellular carcinoma cells by upregulating miR-34a-5p and altering ubiquitination, which activates UL16-binding protein 2 (ULBP2) signaling, promotes infiltration and activation of NK cells, and inhibits tumor progression [88].

## 4. TIME and cisplatin resistance

More than 60% of patients who receive chemotherapy (e.g. neoadjuvant chemotherapy) are resistant to cisplatin [89]. The development of cisplatin resistance primarily depends on three intracellular adaptive mechanisms: decreased uptake or increased efflux of the drug, inactivation of the drug, or activation of DNA damage repair pathways [90–95]. However, recent evidence has shown that the TIME also plays a significant in the development of cisplatin resistance, thereby affecting its therapeutic efficacy [96,97]. The immunological factors associated with cisplatin resistance have been discussed in the following section.

### 4.1. Tumor-associated macrophages (TAMs)

The function of TAMs is strongly correlated to cisplatin resistance [98], and therapies targeting TAMs can potentially sensitize tumor cells to cisplatin [99]. A preclinical study showed that TAMs can induce cisplatin resistance in cancer stem cells (CSCs) by secreting IL-6 [100]. On the other hand, cisplatin induces production of type I IFN, which then binds to the IFN receptor on TAMs and inhibits colony-stimulating factor 1 receptor (CSF-1R) [99]. CSF1R is a type III protein tyrosine kinase receptor that mediates polarization of TAMs to the pro-tumorigenic M2 phenotype [101]. PLX, the first CSF1R inhibitor approved by the FDA [102], prevents M2 polarization of the TAMs and enriches the anti-tumor M1 phenotype [103]. Therefore, CSF1R inhibitors can overcome resistance to cisplatin by modulating the polarization of TAMs [104,105]. In one preclinical study, TAMs increased the resistance of bladder cancer cells to cisplatin by activating the β-catenin/mTOR/CDK6 signaling pathway, which promoted their transition to M2-like macrophages [106]. Another study showed that TAMs in gastric cancer transitioned to the M2-like phenotype through STAT3 activation [107]. In addition, TGF-β1 secreted by M2-like TAMs enhances tumor immune escape and drug resistance by activating the TGFβ1-Smad2/3 pathway [108]. TAMs have also been shown to promote cisplatin resistance in tumor cells by enhance WTAP-mediated N6-methyladenosine RNA methylation through the CXCL16/CXCR6 axis [109]. Interestingly, cisplatin can mediate development of drug resistance through M2-like polarization of TAMs by upregulating IL-34. Consistent with this, IL-34 inhibitors can reverse cisplatin resistance and improve anti-tumor efficacy by inhibiting the M2-like TAMs [110]. Overall, these studies indicate that obstructing the polarization of M2-like TAMs can amplify the response to cisplatin.

### 4.2. Cytotoxic T lymphocytes (CTLs)

Increased peri-tumor infiltration of CTLs can significantly improve the response to chemotherapy, resulting in favorable prognosis [111, 112]. Preclinical studies have shown that GSH secreted by cancer-associated fibroblasts (CAFs) inhibits the nuclear accumulation of cisplatin in ovarian cancer cells, leading to chemoresistance [113]. CTLs can reduce the intra-tumoral GSH content by up-regulating γ-glutamyltransferase and promoting IFN-γ secretion from T cells *via* JAK/STAT1 activation, which sensitizes tumor cells to cisplatin-induced apoptosis [113]. On the other hand, cisplatin-resistant tumor cells induce apoptosis of CTLs by secreting plasma gelsolin (pGSN) and exosome-a type of small extracellular vesicle (sEV), which leads to a decrease in IFN-γ secretion and renders the tumor cells resistant to cisplatin-induced apoptosis [114]. However, increased IFN-γ expression can indirectly trigger apoptosis of CTLs by upregulating PD-L1 on tumor cells [115]. A preclinical study showed that cisplatin down-regulates the transcription factor c-Myc in human ovarian carcinoma cells by inhibiting miR-145, which increases PD-L1 expression and leads to the apoptosis of CTLs, eventually resulting in cisplatin resistance [116]. Furthermore, bladder cancer cells with low methionine content inhibit the infiltration and activation of CTLs, leading to cisplatin resistance [117]. Thus, CTLs act synergistically with cisplatin, and their increased infiltration and activation portends favorable prognosis as well as greater sensitivity to cisplatin.

### 4.3. Cancer-associated fibroblasts (CAFs)

CAFs promote tumorigenesis by remodeling the extracellular matrix, and their association with cisplatin resistance has been extensively studied [118,119]. Preclinical studies have shown that cisplatin promotes exosome secretion by the CAFs, which can inhibit ferroptosis in gastric cancer cells by reducing the accumulation of lipid-ROS, resulting in increased resistance to cisplatin [120]. Furthermore, CAFs secrete chemokines, IL-6, IL-8, IL-11, insulin-like growth factor 1 and TGF-β, which promote immune escape and cisplatin resistance [121]. In one preclinical study, CAFs induced cisplatin resistance in bladder cancer cells through exosomal miR-146a-5p [122], which activated the STAT3 and mTOR signaling pathways by targeting ARID1A and PRKAA2 [122]. Another preclinical study showed that annexin A3 (ANXA3) overexpression in CAFs activated the JNK pathway, which inhibited cisplatin-induced apoptosis in lung cancer cells [123]. In addition, CAFs can also induce cisplatin resistance in bladder cancer cells by activating IGF-1/ERβ signaling and upregulating the anti-apoptotic gene Bcl-2 [124]. Altogether, these studies show that CAFs can enhance resistance of tumor cells to cisplatin.

### 4.4. Other factors

The hypoxic conditions in tumor tissues enhance chemoresistance by promoting stem cell chemotaxis and increasing the expression of multidrug transporter proteins in tumor cells [125–127]. In addition, the aberrant tumor vasculature impairs nutrient supply to the tumor cells, resulting in the metabolic reprogramming to glycolysis [128,129]. The high amounts of lactic acid produced during glycolysis lowers the pH of the tumor microenvironment, which increases drug efflux from the tumor cells and induces chemoresistance [130]. Cell-cell interactions in the TIME are also key determinants of cisplatin resistance [131]. For instance, the interactions between CSCs and immune cells can inhibit the anti-tumor effects of cisplatin and induce resistance [132]. In addition, stromal cells and tumor cells interact to modify the extracellular matrix (ECM) and secrete growth factors that support tumor angiogenesis, inhibit anti-cancer immune responses, and induce cisplatin resistance [133].

## 5. Combination therapies of cisplatin involving the TIME

Cisplatin-based chemotherapy is typically the initial treatment of choice for the majority of cancer patients [11,134]. However, most cancer patients treated with cisplatin alone have a poor prognosis, and may experience the toxic side effects of cisplatin or eventually develop resistance. Several combination therapies have been developed that can overcome these limitations [14] and significantly improve patient survival compared to cisplatin monotherapy [135]. In this section, we have discussed the impact of combining cisplatin with immunosuppressants and lipid metabolism inhibitors on the TIME (Table 1).

**Table 1.** Overview of immunosuppressants and lipid metabolism inhibitors used in combination with cisplatin. The combination of cisplatin and inhibitors can alter the TIME and signaling of many types of tumor cells, improving the efficacy of cisplatin.

Targets	Impact on the outcome	Ref
CD8T, TNF-α, and JAK-STAT signals	Up	137
CD8T, cGAS-STING signals	Up	138
CD8T, CXCR3 / CXCL10	Up	139
	The patient’s prognosis improves	140
CD8T, cGAS-STING signaling, MHC-I, Calreticulin	Up	141
	The patient’s prognosis improves	142
CD8 + Cxcr3 + IFN-γ+ T cells	Up	143
Cyclin B1	Down	144
Fatty acids β oxidation, glutamine metabolism, nucleotides and glutathione synthesis	Down	145
GSH/GSSG、GPX4	Down	146

### 5.1. Immune checkpoint inhibitors (ICIs)

ICIs have a favorable safety profile compared to cytotoxic drugs and may significantly improve the prognosis of cancer patients when used in combination with cisplatin. Several clinical trials are underway to test the efficacy of ICIs in cisplatin-resistant and ICI-sensitive cancer patients [136]. For example, cisplatin-resistant urothelial carcinoma patients were treated with the combination of PD-L1 inhibitor durvalumab and CTLA-4 inhibitor tremelimumab, and 37.5% of the patients achieved complete pathological remission, while 58% achieved stage reduction to pT1 or lower [137]. In a phase II trial conducted on cisplatin-resistant bladder cancer patients, neoadjuvant treatment with the anti-PD-1 antibody pembrolizumab prior to radical cystectomy effectively reduced tumor progression [138].

The efficacy of ICIs depends significantly on the immune landscape of tumors, including the number and activation status of CD8+ T cells, the presence of other immune cells, and local cytokine signaling [60]. In addition, since ICIs can directly alter the TIME, their effect is also dependent on the interaction between cancer cells and immune cells [60]. Most ICIs that have been developed so far target PD-1 and PD-L1 expressed on the tumor-infiltrating T cells and tumor cells respectively [139]. Disrupting the interaction between PD-1 and PD-L1 neutralizes the inhibitory signals in T cells, and activates the anti-tumor immune response [140]. Monoclonal antibodies targeting PD-1 and PD-L1 have been successful in treating a wide range of cancer types [141], and have demonstrated durable therapeutic activity in several clinical trials [142–144].

#### 5.1.1. PD-1 inhibitors

**Figure 3.**
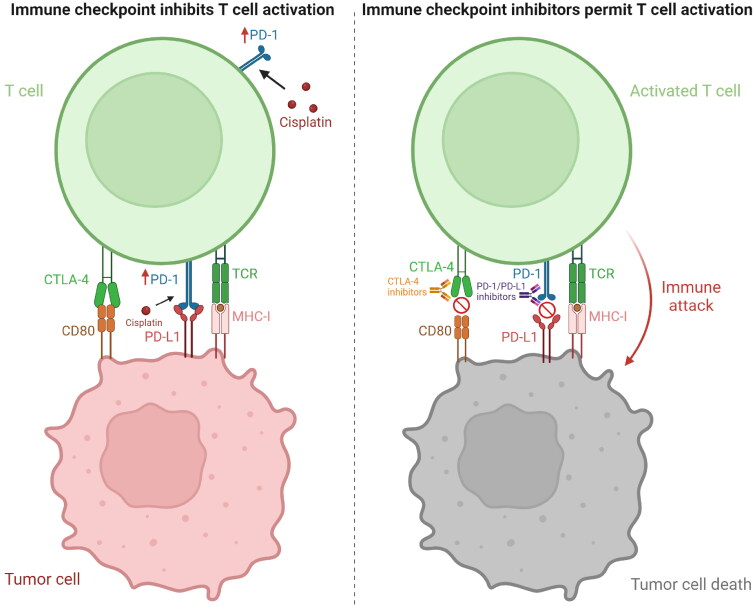
Effect of cisplatin and immune checkpoint inhibitors on immune checkpoints. T cells recognize the MHC-I molecules through their surface TCR, leading to T cell activation. However, PD-1 and CTLA-4 on T cells bind to PD-L1 and CD80 on tumor cells to inhibit T cell activation. In addition, cisplatin resistance can lead to upregulation of PD-1 to inhibit T cell activation and thus promote tumor cell proliferation. PD-1/PD-L1 and CTLA-4 inhibitors inhibit the binding of PD-1 to PD-L1 and CTLA-4 to CD80, thereby promoting T cell activation and tumor cell death. MHC-I: major histocompatibility complex class I, TCR: T cell receptors, PD-1: programmed death receptor 1, PD-L1: programed death ligand 1, CTLA-4: cytotoxic T lymphocyte-associated protein 4, CD80: cluster of differentiation 80. Created with BioRender.com.

PD-1 is primarily expressed on T cells, and its binding with PD-L1 suppresses T cell function and decreases cytokine production [145]. In addition, PD-1 is also expressed in various tumors, such as bladder uroepithelial carcinoma and renal cancer, and blocking the PD-1 pathway confers an overall survival benefit in patients with renal cell carcinoma [146–148]. Cisplatin resistance can promote tumor cell proliferation and suppress CD8+ T cells by upregulating PD-1, rendering it less effective [149]. This suggests that inhibition of PD-1 may reduce cisplatin resistance and improve therapeutic efficacy (Figure 3).

Preclinical trials have shown that using cisplatin in conjunction with PD-1 inhibitors can not only boost the anti-tumor effects of cisplatin but also improve the outcomes of immunotherapy [150]. Furthermore, cisplatin and anti-PD-1 antibodies can synergistically increase CTL infiltration and activation, and promote secretion of anti-tumor cytokines [151,152]. Yu et al. showed that cisplatin induced G2/M checkpoint arrest and apoptosis in kidney cancer cells, and activated the cGAS-STING signaling pathway by increasing the level of cytoplasmic dsDNA [153]. Activation of the cGAS-STING pathway can reverse the immunosuppressive TIME by upregulating type I IFN, and enhance the efficacy of immunotherapy [154]. Furthermore, type I IFN also enhances presentation of tumor antigens by DCs and macrophages, which promotes the infiltration and activation of anti-tumor CD8+ T cells [155]. Cisplatin can augment the therapeutic effect of PD-1 inhibitors by inducing CXCL10 secretion and the intra-tumoral infiltration of CD8+ T cells [156]. Taken together, the combination of cisplatin and PD-1 inhibitors can potentially inhibit cisplatin-resistant tumors and improve therapeutic outcomes.

#### 5.1.2. PD-L1 inhibitors

PD-L1 is an immunosuppressive factor that is expressed on various tumor cells, as well as the immune cells and non-immune cells in the tumor tissues [157]. Upregulation of PD-L1 in the TIME is the most fundamental mechanism driving immune evasion of tumor cells [158], and high PD-L1 expression in cancer patients is usually associated with poor clinical outcomes [159,160]. However, PD-L1 expression is not an accurate predictor of the anti-tumor immune response, since PD-L1-negative tumors can be sensitive to ICI therapy, while PD-L1-positive tumors may respond poorly [161,162]. Currently, PD-L1 inhibitors are approved for treating PD-L1-positive and cisplatin-resistant tumors, and for post-cisplatin maintenance therapy in refractory patients [163]. PD-L1 can also desensitize cancer cells to cisplatin-induced DNA damage by binding to RNA exosomes and protecting the regulatory mRNAs from degradation [164]. Therefore, PD-L1 inhibitors may be more effective against cisplatin-sensitive tumors [165]. In addition, the combination of cisplatin and PD-L1 inhibitors led to a notable decrease in tumor size in a murine lung cancer model and prolonged the survival tumor-bearing mice compared to anti-PD-L1 monotherapy [166].

Preclinical studies have shown that cisplatin increases PD-L1 expression and stability on ovarian cancer cells by activating the cGAS-STING signaling pathway [167], which enhances production of type I IFN and other immunomodulatory molecules, and increases recruitment of DCs and CD8+ T cells [168]. Elevated IFN levels can significantly enhance the recognition and elimination of tumor cells *via* innate and adaptive immune cells, thereby augmenting the effects of immunotherapy [169]. Furthermore, cisplatin has been shown to increase chemoresistance in lung cancer cells by upregulating PD-L1 and inhibiting the tumor-infiltrating lymphocytes through the activation of phosphatidylinositol 3-kinase/protein kinase B pathway [170]. Cisplatin in combination with anti-PD-L1 antibodies can improve the outcomes of immunotherapy in cancer patients [171], and achieve better outcomes against tumors with a high infiltration of CD8+ CD103+ T cells [172].

#### 5.1.3. CTLA-4 inhibitors

CTLA-4 is an immunomodulatory molecule that is primarily induced on the surface of T cells [173]. It competes with CD28 for its co-ligands B7-1 (CD80) and B7-2 (CD86) and bind to them with higher affinity, thereby inhibiting T cell proliferation and function [174]. In addition, CTLA-4 also creates am immunosuppressive milieu in the tumors by promoting the development and function of T-regulatory cells (Tregs), and increasing CD80 and CD86 expression on the DCs [175]. CTLA-4 inhibitors enhance the anti-tumor effects of IL-36 by inhibiting Tregs, resulting in increased proliferation of CD8+ T cells and IFN-γ production, which act synergistically with cisplatin [176].

A recent clinical study showed that the anti-CTLA-4 antibody ipilimumab optimized the response rate in small cell lung cancer patients treated with a combination of cisplatin and etoposide [177]. While PD-1 blockade improved prognosis of cisplatin-treated biliary tract cancer patients, the overall response rate was low [178]. On the other hand, a CTLA-4 inhibitor (clone 9D9) in combination with anti-PD-1 antibody and cisplatin inhibited tumor growth in a mouse model of cholangiocarcinoma [178]. Cisplatin normalized the tumor blood vessels, leading to increased infiltration of CD8+ T cells and upregulation of T cell activation markers IFN-γ and CXCR3, when combined with dual CTLA-4 and PD-1 blockade [178]. Given the synergistic effects of cisplatin and CTLA-4 inhibitors, this combination therapy can improve anti-tumor immune responses and overcome drug resistance, and warrants more preclinical and clinical studies.

### 5.2. Inhibitors of lipid metabolism

**Figure 4.**
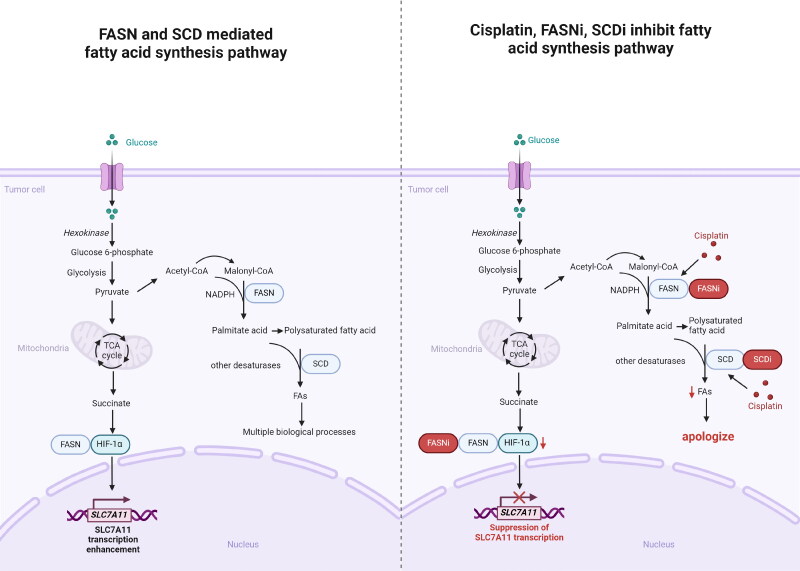
Cisplatin, FASN inhibitors, and SCD inhibitors work together to regulate fatty acid biosynthesis pathways. Glucose is phosphorylated to Glucose-6-phosphate by hexokinase when ingested by tumor cells and enters the glycolytic pathway. The pyruvate produced enters the mitochondria and enters the TCA cycle, and the resulting succinic acid activates HIF-1α. FASN enhances transcription of SLC7A11 by binding to HIF-1α. FASN inhibitors block this process, inhibiting the transcription of SLC7A11. In addition, acetyl-CoA produced by pyruvate is converted to malonyl-CoA. Using NADPH as a reducing cofactor, FASN catalyzes the synthesis of palmitic acid by acetyl-CoA and malonyl-CoA, which then synthesize FAs to participate in a variety of biological processes. Cisplatin and FASN inhibitors reduce FASN expression, reduce FAs synthesis and increase apoptosis of tumor cells. Cisplatin and SCD inhibitors also reduce FAs synthesis and increase apoptosis in tumor cells. TCA: tricarboxylic acid, FASN: Fatty acid synthase, FASNi: Fatty acid synthase inhibitors, SCD: Stearoyl-CoA desaturase, SCDi: Stearoyl-CoA desaturase inhibitors, HIF-1α: Hypoxia-inducible factor-1α, SLC7A11: Solute carrier family 7 member 11, NADPH: Nicotinamide adenine dinucleotide phosphate, FAs: fatty acids. Created with BioRender.com.

Lipid metabolism, particularly fatty acid (FA) synthesis, is crucial for cell membrane biosynthesis, production of signaling molecules, and energy storage [179]. Most immune cells, including regulatory T cells and memory T cells, rely on lipid oxidation as the primary energy source [180]. In addition, the hypoxic tumor microenvironment prevents utilization of glucose in FA synthesis, which increases the dependency of tumor cells on exogenous lipid uptake [181]. However, if exogenous lipids are also in short supply, the cells may synthesize FAs de novo to ensure sufficient intracellular lipid content. Cisplatin increases lipid deposition in tumor cells, which in turn increases ROS production and induces apoptosis [182]. ROS accumulation in also increases lipid peroxidation in the cell membrane and triggers ferroptosis [183]. Therefore, the combination of cisplatin and lipid metabolism inhibitors is a promising new strategy for the treatment of cisplatin-resistant tumors (Figure 4).

#### 5.2.1. Fatty acid synthase (FASN) inhibitors

FASN is an oncogenic protein that protects tumor cells against cisplatin-induced apoptosis [184]. Thus, the combination of FASN inhibitors and cisplatin can synergistically inhibit tumor growth and metastasis, and improve patient prognosis. FASN inhibitors are particularly effective against cancers with dysregulated lipid metabolism, and target multiple anabolic and oncogenic signaling pathways [185].

In a recent preclinical study, a FASN inhibitor promoted T helper cell 1 (Th1) infiltration and activation, increased IFN-γ production, and induced apoptosis of tumor cells [186]. Another study showed that FASN inhibitors decreased aberrant lipid accumulation in the TIME, which promoted the activation and accumulation of DCs as well as anti-tumor T cells, and re-sensitized tumor cells to immunotherapy [187]. Furthermore, FASN inhibitors can reverse the immunosuppression in the tumor microenvironment by upregulating cytotoxicity markers in NK cells and CD8+ T lymphocytes [188]. However, FASN inhibitors can also induce drug resistance and immune evasion by suppressing the PI3K-AKT-mTOR pathway and inhibiting T cell activation and function. Consequently, the combination of FASN inhibitors and T cell activators may enhance the response of tumor cells to FASN inhibitors [189]. In addition, cisplatin also decreased FASN expression and increased apoptosis of breast cancer cells, suggesting that the combination of FASN inhibitors and cisplatin may improve the prognosis of cancer patients [184]. FASN inhibitors blocked the FASN-HIF1α-SLC7A11 pathway signaling in hepatocellular carcinoma cells, and delayed tumor growth by inducing apoptosis and necrosis [190]. In addition, FASN inhibitors also downregulated the cell cycle protein cyclin B1 in oral squamous cell carcinoma cells and sensitized them to cisplatin [191]. The combination of FASN inhibitors and cisplatin also decreased fatty acid metabolism, glutamine metabolism, nucleotide and glutathione biosynthesis, and fatty acid β-oxidation in tumor cells, resulting in cell cycle arrest and extensive cell death [192].

#### 5.2.2. Stearoyl coenzyme a desaturase (SCD) inhibitors

SCD is expressed in the endoplasmic reticulum and regulates the synthesis and metabolism of unsaturated fatty acids [193]. It can promote the proliferation and invasiveness of cancer cells by activating the Wnt signaling pathway, and contribute to their metabolic reprogramming by producing monounsaturated FAs that regulate the AKT, AMPK and NF-kB pathways [194]. Combining SCD inhibitors with cisplatin may suppress the proliferation and metastasis of cancer cells through synergistic effects and improve the anti-tumor effects of cisplatin.

Preclinical studies have shown that SCD gene deletion inhibits secretion of monounsaturated FAs, resulting in cGAS-STING activation [195], which in turn promotes type I IFN signaling in the TIME and upregulates IFN-stimulated genes (ISGs) [196]. Type I IFN and ISGs enhance the anti-tumor innate and adaptive immune responses, the augment the effects of immunotherapy [197]. Furthermore, SCD1 inhibitors enhance CCL4 production by cancer cells through the inactivation of Wnt/β-catenin signaling, which promotes recruitment of DCs into the tumors, and increases CD8+ T cells infiltration and activation [198]. Another preclinical study demonstrated that inhibition of SCD1 upregulated acetyl coenzyme A acetyltransferase 1 (ACAT1), resulting in increased infiltration and activation of CD8+ T cells [199]. In addition, the combination of cisplatin and SCD1 inhibitors synergistically impaired lipid metabolism in tumor cells, which decreased tumor invasiveness and improved patient prognosis [200]. Therefore, combination therapy of cisplatin and SCD inhibitors may improve the outcomes against cisplatin-resistant tumors.

### 5.3. Nanoparticles (NPs)

Nanomedicine offers a new strategy for overcoming the chemoresistance of tumor cells. The main advantages of using nanocarriers for cisplatin delivery include selective targeting of tumor cells, reduced systemic toxicity, enhanced cellular uptake, and increased accumulation of cisplatin within tumor cells [201]. NPs enter cells through endocytosis, and can therefore effectively deliver cisplatin into drug-resistant tumor cells [202]. In addition, the small size and stability of the NPs increase the likelihood of accumulation at tumor sites, thereby improving systemic diffusion of of the drug cargo [203]. Therefore, NPs-mediated delivery of cisplatin can significantly reduce side effects, especially nephrotoxicity and neurotoxicity, and improve treatment outcomes compared to that of free cisplatin [204].

ROS-responsive NPs consisting of cisplatin, camptothecin (CPT), ROS-sensitive polymer and mPEG2k-DSPE accumulated in the tumor tissues in a preclinical model of colorectal cancer, and released cisplatin and CPT in the target regions [66]. The targeted drug release induced DNA damage in the tumor cells, activated the cGAS-STING pathway, and increased the infiltration of DCs and CD8+ T cells in the TIME, resulting in enhanced anti-tumor immune response [66]. In addition, ROS-responsive NPs can inhibit the activity of glutathione S-transferase (GST), reduce GSH-mediated cisplatin resistance, and improve the chemotherapeutic effect of cisplatin [205]. ROS-responsive NPs triggered ROS production in prostate tumor cells upon stimulation with 808 nm near infra-red radiation, resulting in type II ICD that induced a stronger anti-tumor immune response [205]. Furthermore, cisplatin conjugated to Au NPs promoted the infiltration of CD8+ T cells and DCs in the lung cancer TIME, and enhanced the anti-tumor immune response and effects of cisplatin [206]. Taken together, the combined use of cisplatin and NPs can amplify the immune response in TIME, improve cisplatin resistance, and enhance chemotherapeutic effects.

## 6. Conclusions and future directions

Cisplatin affects the immune cells in the tumor cells and conversely, immune cells aid in the development of cisplatin resistance through complex mechanisms. Studies show that cisplatin can enhance the activation and maturation of TAMs, CTLs, DCs, and NK cells, thereby strengthening anti-tumor immune response. In addition, cisplatin also inhibits the immunosuppressive cells like Tregs and MDSCs, which further augments immune-mediated clearance of tumor cells. Although several studies have explored the role of cisplatin in the TIME, its specific mechanism of action still needs further investigation.

Nevertheless, the common side effects of cisplatin, including nephrotoxicity, ototoxicity, hepatotoxicity, cardiotoxicity and neurotoxicity, limit its dosage and frequency of use. In addition, development of cisplatin resistance also hinders its therapeutic efficacy. Combining cisplatin with immune checkpoint blockers (such as PD-1/PD-L1 or CTLA4 inhibitors), lipid metabolism disruptors (like FASN inhibitors and SCD inhibitors) and NPs can reduce the dose of cisplatin, reverse cisplatin resistance and reduce its side effects, and improve patient outcomes. However, a combination of drugs may lead to cumulative drug toxicity and worsen the side effects of treatment. Therefore, continued research into cisplatin as well as the TIME, and the development of new combination therapy strategies are essential to overcome the existing limitations and challenges.

## Data Availability

Data sharing is not applicable to this article because it is a narrative review and as such, no new data were created or analyzed in this study.
